# Ethical challenges related to elder care. High level decision-makers' experiences

**DOI:** 10.1186/1472-6939-8-3

**Published:** 2007-04-10

**Authors:** Anna-Greta Mamhidir, Mona Kihlgren, Venke Sorlie

**Affiliations:** 1 Department of Neurobiology, Caring Sciences and Society, Karolinska Institute, Stockholm, Sweden; 2 Centre for Nursing Science, Örebro University Hospital, Örebro, Sweden; 3 Bodo University College, School of professional studies, Center for Practical Knowledge, Norway

## Abstract

**Background:**

Few empirical studies have been found that explore ethical challenges among persons in high public positions that are responsible for elder care. The aim of this paper was to illuminate the meaning of being in ethically difficult situations related to elder care as experienced by high level decision-makers.

**Methods:**

A phenomenological-hermeneutic method was used to analyse the eighteen interviews conducted with political and civil servant high level decision-makers at the municipality and county council level from two counties in Sweden. The participants worked at a planning and control as well as executive level and had both budget and quality of elder care responsibilities.

**Results:**

Both ethical dilemmas and the meaning of being in ethically difficult situations related to elder care were revealed. No differences were seen between the politicians and the civil servants. The ethical dilemmas mostly concerned dealings with extensive care needs and working with a limited budget. The dilemmas were associated with a lack of good care and a lack of agreement concerning care such as vulnerable patients in inappropriate care settings, weaknesses in medical support, dissimilar focuses between the caring systems, justness in the distribution of care and deficient information. Being in ethically difficult situations was challenging. Associated with them were experiences of being exposed, having to be strategic and living with feelings such as aloneness and loneliness, uncertainty, lack of confirmation, the risk of being threatened or becoming a scapegoat and difficult decision avoidance.

**Conclusion:**

Our paper provides further insight into the ethical dilemmas and ethical challenges met by high level decision-makers', which is important since the overall responsibility for elder care that is also ethically defensible rests with them. They have power and their decisions affect many stakeholders in elder care. Our results can be used to stimulate discussions between high level decision-makers and health care professionals concerning ways of dealing with ethical issues and the necessity of structures that facilitate dealing with them. Even if the high level decision-makers have learned to live with the ethical challenges that confronted them, it was obvious that they were not free from feelings of uncertainty, frustration and loneliness. Vulnerability was revealed regarding themselves and others. Their feelings of failure indicated that they felt something was at stake for the older adults in elder care and for themselves as well, in that there was the risk that important needs would go unmet.

## Background

For more than a decade, ethical challenges in health care organisations have been reported [1]. The older population in Sweden is increasing as in the rest of the developed countries. During the last twenty years the number of persons aged 80 or older has increased 81% from 263,000 to 476,000 [2]. The cost of providing care for this group has posed an economic problem at the local and national levels [3]. Furthermore, the number of older adults with origins from other countries is increasing. As a result new demands are being placed on those responsible for elder care [3]. The responsibility for elder care is shared between the local municipalities and the county councils. The main responsibility for elder care lies with the local municipalities and when acute care is required the responsibility is shifted to the county councils. Employment of the physicians occurs through the county councils, while the nurses and aides can be employed by either one of the organisations.

Following older adult health trends is important in order to prepare for future medical and social care needs [4]. Reports of problems in fulfilling basic needs in elder care have been reported due to the over burdened system [5]. One of the problem areas cited concerns nutritional deficiencies [6]. Increased care needs together with declining budgets contribute to ethical challenges in the health care system [7]. Health care workers experience ethical dilemmas when they find it difficult to know what is the right and good thing to do [8].

Ethical dilemmas occur when there are at least two conflicting choices of how to deal with something and neither leads to a positive outcome [9]. A study that involved physicians in ethically difficult situations has shown that they can have doubts as to which actions for the patients are the best ones to take in specific situations [10]. When nurses feel they are not able to fulfil their professional ideals in ethically difficult situations, they sense their moral self-image can be threatened, which causes them distress [11]. These medical and nursing care issues can be understood from an action and relational ethics perspective. The action ethics perspective concerns the question, what a person should or ought to do in ethically difficult situations and why. In this perspective the ethics are often centred on ethically difficult dilemmas and on the making of a decision [9]. A relational ethics perspective refers to the reflection on the challenges encountered in person's relationship with others and on how to fulfil social roles and obligations in a good way, as a human being, a colleague or a high level decision-maker (HDM). How a person can best meet the challenges they confront in a situation or a relationship and how they are involved, is the question raised with this perspective. That which makes a person good is not only their traits but also the characteristics of the situation and the relationships in it. It has been emphasised that both perspectives are needed at the same time [9].

In the public health care system, HDMs i.e. the politicians and appointed civil servants have both budget and quality of elder care responsibilities. Their position requires them to make decisions that seem to a large extent have to do with the action ethics perspective. There are many stakeholders i.e. all those effected by the HDMs' decisions; the patients, the relatives and the different health care providers and givers.

Previous studies have focused on ethical difficulties among health care professionals in various situations [10-14]. Few empirical studies have been found that explore ethically difficult situations confronted by the HDMs responsible for elder care. The present study, which is a part of a larger project, included interviews with HDMs concerning these aspects. The aim of this paper was to illuminate the meaning of being in ethically difficult situations related to elder care as experienced by the HDMs.

## Methods

### Participants and setting

High level decision-makers at the municipality and county council level from two counties in Sweden were invited to participate. The HDMs were elected politicians and appointed civil servants at a planning and control as well as executive level that had both budget and quality of elder care responsibilities. The selection of the participants was made from a compiled list of municipal and county council HDM names from the two counties. Names were drawn until there were a total of 18 willing participants. The first 18 asked were willing to participate with the result being nine politicians and nine civil servants. They ranged in age from 43 to 66 years, had held their positions between one to 20 years and 13 were female.

Participation was voluntary and all 18 completed their interviews and could end them at any time without giving a reason. Written consent was given after both verbal and written information was provided. Confidentiality was assured and there would be no possibility to trace the findings to the participants. The Regional Research Ethical Committees approved this study (99310-17).

### Data collection

#### Interviews

The HDMs chose the location for the interviews, which was often their office. They were asked *"Please tell me about one or more of the ethically difficult situations regarding elder care that you have experienced in your position*." The concept of an ethically difficult situation was not defined, which allowed the interviewees to discuss what they considered to be ethically difficult. Allowing the HDMs to make their reflections without interruption was done so that the narratives would be as rich as possible in content. Follow-up questions concerning the HDMs' thoughts, feelings and actions such as *"Tell me more about that" *or *"What do you mean by that" *were asked when the interviewer wanted them to elaborate further or give clarification [15]. The individual interviews were tape-recorded and transcribed verbatim. Notes were also taken during the interviews to help with orientation and understanding in the analysis phase and included non-verbal communication such as laughter and long pauses.

### Data analysis

#### Interpretations

A phenomenological hermeneutic method [16] was used to analyse and interpret the interview text. Several other authors have used this method [8,12,17,18] that is useful when attempting to elucidate the meaning of a lived experience through the interpretation of an individual's narrative. The phenomenological hermeneutic analysis process consists of three phases: the *naïve reading*, one or more *structural analyses *and a *comprehensive understanding*. The analysis process constitutes a dialectal movement between the whole and the parts of the text and between understanding and explanation [19].

In the first phase a *naïve reading *of the transcribed interviews was done with an open-mind to gain a first impression of the text as a whole concerning what the HDMs' experienced as being ethically difficult situations. The naïve reading indicated the direction for the subsequent analyses. The narratives were shown to include both illumination of ethical dilemmas and the HDMs' experiences of being in ethical challenges. In the second phase *structural analyses *were performed, which aredetailed analyses of the text in order to explain the parts and validate or invalidate the initial understanding gained from the naïve reading. The text was divided into meaning units that were then condensed, abstracted and structured into sub-themes and themes [16]. A meaning unit can be a part of a sentence, a whole sentence or a paragraph. The sub-themes and themes are presented under the heading Results. A *comprehensive understanding *was developed in the third phase in which the authors' pre-understandings, the naïve reading, the structure analyses and relevant literature were taken into account. This is addressed under the heading Discussion. All authors took part in the analyses until agreement over the interpretation and findings were considered satisfactory.

The interviews with the HDMs were rich and detailed. Early in the analysis phase it became evident that the HDMs did not differentiate in their narrations between the action ethics perspective and the relational ethics perspective previously described [9]. When telling about ethically difficult situations, it is natural not to separate between these two perspectives since these type of dilemmas and the feelings of being in such situations are often expressed at the same time. This analytic distinction has previously been used in other studies as a basis for the structure and the presentation of the results [13,14]. We decided to separate the analysis and the presentation of the results in the same manner since it gives a possibility to reveal the complexity of the different ethical perspectives in the narratives [9,11]. This separation has resulted in two parts under the heading Results, where the first part addresses the ethical dilemmas related to elder care confronted by the HDMs and the second part addresses their experiences of the meaning of being in ethically difficult situations related to elder care.

## Results

Several ethical dilemmas and ethical challenges confronted the HDMs associated with elder care. No differences could be seen between the politicians and the civil servants or between the municipality and county council levels, with the method used, regarding the phenomenon of ethical dilemma or the meaning of their experiences of being in ethically difficult situations.

### Ethical dilemmas

The themes and sub-themes developed are presented in Table 1. Quotations that illuminate the themes and sub-themes are included in the text.

**Table 1:** Overview of themes and sub-themes that developed from the structural analysis of interviews with the high level decision-makers about ethical dilemmas.

**Themes**	**Sub-themes**
***Lack of good care***	*Vulnerable patients in inappropriate care settings* *Poor and disrespectful interactions* *Weaknesses in medical support*
***Lack of agreement concerning care***	*Abandoned ideals vs. budget realities* *Dissimilar focuses between the caring system* *Justness in the distribution of care and deficient information*

#### Lack of good care

##### Vulnerable patients in inappropriate care settings

The HDMs reflected on how the governments good intentions which are aimed at helping older adults remain in their private residences as long as possible have led to a lack of care and ethical dilemmas. They felt many of the older adults being cared for at home have illnesses that require extensive and at times advanced care that can be difficult to provide for there. According to them, ethical dilemmas have been accentuated with last decade's economic reductions and the decreased number of beds in sheltered housing facilities. An ethical dilemma that was mentioned was the lack of available residences causing patients with dementia to be placed in inappropriate settings. "It is difficult when persons with dementia disease live in mixed care settings with others that don't have it. It is difficult for everyone, but especially for those with dementia since they are so vulnerable".

##### Poor care and disrespectful interactions

The HDMs told of the ethical dilemmas associated with the strained conditions in elder care. They said that caregivers working in the homes worked in a hurried task oriented manner that did not address the individual patient's needs. According to them, the caregivers have been reported as being in such a hurry that they put meals in the refrigirator without noticing there were several still in there uneaten. Receiving information from mass media or upset relatives of insufficinet care due to poor and sometimes disrespectful interactions on the part of the caregivers, was also highlighted. This was distrubing since the HDMs expected health care professionals to know how to interact with patients.

##### Weaknesses in medical support

Ethical dilemmas related to the lack of good medical care was said to occur when the ill patients were poorly assessed or received poor medical service due to a continuous shortage of physicians. They spoke of the physician shortage in elder care causing unnecessary referrals of older adult patients to the hospitals and felt that daily caregivers often lacked the competence to make referral decisions. The Elder-reform of 1992 with its strong emphasis on the social aspects resulted in a weakening of the medical priorities in elder care, according to the HDMs.

#### Lack of agreement concerning care

##### Abandoned ideals vs. budget realities

The HDMs told of the ethical dilemmas related to the demands of maintaining a balanced budget with the ever-increasing needs in elder care. Short-term budget solutions, downsizing of sheltered housing facilities and relatives having to assume more responsibility, were named in association with such dilemmas. The HDMs experienced ethical dilemmas when they felt forced to abandon their vision of ideal elder care to budget realities.

##### Dissimilar focuses between the caring systems

The HDMs spoke of the ethical dilemmas associated with a care system they felt did not always meet the patients' needs. "We have built up a system that seems to mostly benefit the system itself, the employees or possibly those in power". They also said that the dissimilar focuses regarding elder care between the county council and municipal health care systems contributed to ethical dilemmas. "The needs of the whole person are not looked after, it's about all the levels of elder care focusing on the older adults' possibilities and strengthening them. If we all had the same focus we could get over a few of the hurdles. The established structure can contribute to ethical problems". They thought that a change would not occur until a new generation assumed its place in the profession, in politics, and in the administration.

##### Justness in the distribution of care and deficient information

Ethical dilemmas associated with the problems within the system that made it difficult to distribute resources in a just manner were described. The HDMs told of the old and chronically ill that should have priority, but as there are others i.e. the younger and less ill that also need access to health care, there were often conflicts and strong voices involved. HDMs found it difficult with incomplete information and a poor reporting system that did not give them a clear description of individual or organisational needs. They spoke of the ethical dilemmas encountered with a system that allows for insufficient input and inexplicit guidelines.

### The meaning of being in ethically difficult situations

The themes and sub-themes developed are presented in Table 2. Quotations that illuminate the themes and sub-themes are included in the text.

**Table 2:** Overview of themes and sub-themes that developed from the structural analysis of interviews with the high level decision-makers' experiences of being in ethically difficult situations.

**Themes**	**Sub-themes**
***To be in a high position***	*Aloneness and Loneliness* *Uncertainty* *Lack of confirmation* *Scapegoat*
***To be in an exposed position***	*Bombarded* * Threats*
***To be strategic***	*Avoiding difficult decisions* * Handling different opinions*
***To live with divided feelings***	*Loyalties vs. own conviction*

#### To be in a high position

##### Aloneness and Loneliness

HDMs in their high positions with heavy responsibility experienced emotional distress especially in situations associated with budget cuts. "I have assumed the responsibility; that is part of the job. I get paid for it, but it still feels heavy. I often lay sleepless, I get frustrated, I feel alone, and we don't talk with each other that much about our frustrations. But this is a job that has to be done, if not by myself by someone else." They felt that aloneness is something that goes with the job. A lack of support could intensify their feeling of loneliness since they could not depend on anyone else when things got tough. "It's lonely at the top but that's how it is with the ultimate responsibility."

##### Uncertainty

The HDMs stated that it was ethically challenging when they had to make decisions they were not certain of or when the consequences for the older patients were unforeseeable and unclear. Living with uncertainty was something they felt they could not escape from. "If I'm unable, then I have to resign". Receiving reports of insufficient patient care was also challenging since the HDMs felt uncertain they could actually trust the system.

##### Lack of confirmation

HDMs felt ethically challenged when there was a lack of confirmation and that their health pays a high price for it. They found it difficult when they often heard that things were not working out well and seldom received any positive feedback. The HDMs told of how they could get some confirmation at home but there were limits to that.

##### Scapegoat

In their top positions where difficult decisions had to be made, the HDMs said they felt vulnerable and were easily targeted as a scapegoat. They told how many persons quit or got fired. Preserving a good relationship with the media was mentioned as being important but they found it difficult when they were hunted down by the mass media.

#### To be in an exposed position

##### Bombarded

The HDMs often felt exposed and bombarded by many different factions and found dealing with them all to be ethically challenging. They felt pressure from all sides, from the public, from the different health care professionals and the rules and regulations that have to be followed. The personal demands they place on themselves also bombarded them at times, they said. The HDMs felt that these ethical challenges could be met by finding positive aspects in their work. "The job gives me satisfaction and a person feels important. The driving force is that you want to improve health care".

##### Threats

The HDMs spoke of threats made by the public, which became most acute when they had made decisions that called for the closing of healthcare facilities. "Threats, in fact it's unpleasant being in this position, just standing there and taking it".

#### To be strategic

##### Avoiding difficult decisions

"It won't be long before the national politicians will be forced to take a stand on where the lines for elder care are to be drawn. A dialogue has been going on for a while with the professionals in which they have asked us to tell them what they should and should not do, but we haven't done that yet. This is a difficult area to address and it's only human to take up the most difficult issues last". According to the HDMs, the national allocation of resources between the different groups in the health care system has not been discussed in an open public debate. They said it was important to understand the processes involved when working in a socialised health care system.

##### Handling different opinions

The HDMs stated that they constantly had to deal with different opinions. Opinions could come from the different public interest groups or from different administrative entities within the health care system.

#### To live with divided feelings

##### Loyalty vs. own conviction

Making decisions that would have negative consequences for elder care were mentioned as being ethically challenging since it left them with divided feelings. The HDMs felt they had failed in their mission to provide good health care when their loyalty to their job forced them to make reductions. They also said that their position steers their actions since that is what is expected of them and that they are a part of a system with many actors involved.

## Discussions and conclusion

The HDMs in this study, revealed both ethical dilemmas and their experiences of being in ethically difficult situations related to elder care. They were directly or indirectly involved with the dilemmas. The ethical dilemmas revealed were associated with a lack of good elder care and a lack of agreement concerning care. These issues addressed for example vulnerable patients in inappropriate care settings, weaknesses in medical support, dissimilar focuses between the caring systems as well as justness in the distribution of care and deficient information. The HDMs' experiences of being in ethically difficult situations were associated with their exposed situations, feelings of having to be strategic and having to live with divided feelings. These issues addressed aspects such as aloneness and loneliness, uncertainty, lack of confirmation, risk of being threatened or of becoming a scapegoat. Avoidance of difficult decisions and loyalty vs. own conviction was also highlighted.

Lack of good care such as inappropriate care settings for patients with dementia disease were seen as ethically problematic. Chronically ill individuals are to be met with dignity and should have priority in the health care system [20,21]. Future conflicts related to the extensive care needs of a growing older population together with decreased resources are well known [22]. Ensuring good residences for the most vulnerable patients will inevitably be one of the dilemmas on the HDMs agenda.

Weaknesses in medical support due to a continuous shortage of physicians in elder care were expressed as ethically troublesome. Unnecessary transfers of older adults to the hospital were mentioned as a result of this situation. High transfer rates among older adults living in sheltered housing facilities to the emergency departments have been reported, but more analysis was recommended [23]. The shortage of physicians in elder care and current recruiting difficulties can originate from several causes but are still problems the HDMs are obligated to resolve. Physicians have ranked caring for older patients last in the area of prestige [24]. This ranking indicates the degree of esteem the physicians, health care services and society in general seem to have towards older adults [12].

Dissimilar focuses and lack of structure and agreement between the municipalities' and the county councils' organisations responsible for elder care in Sweden were reported in our study as being a cause of ethical dilemmas. This is comparable to what Thompsen [25] called "the problem of many hands", which means that when many officials contribute to decisions and policies of government it is difficult to identify who is morally responsible for the political outcomes. A society can be referred to as being unethical if it has vague policies. An example mentioned is a health care system that lacks structure and policy [26]. In our study it was empathized that lack of structure is a factor contributing to ethical difficulties. It is reasonable to assume that the HDMs are to a large extent responsible for handling the problems related to the organisational structure deficiencies especially since a system can interfere with the understanding of what is right and fair and can thereby be a source of ethical dilemmas or can prevent them [26]. In a system lacking policy and structure, it can be difficult to act ethically even if people have the intention to do so [26].

Lack of agreement concerning care was related by the HDMs as the incongruence between health care needs and the budget. This situation has caused relatives to assume a greater amount of responsibility, which is something they question to some extent the appropriateness of. Relatives are already assuming much responsibility in elder care and many of those doing so are the spouses [27]. Additionally, the reduction of sheltered housing facilities in several municipalities has been rapid and there are few studies that investigate the consequences of these changes [28].

Dealing with the justness in the distribution of care between different groups was referred to in our study as a source of ethical dilemmas. This justness was associated with the difficulties of prioritising and fairly providing for the care needs of the different groups while still remaining within the budget. When preconditions associated with priority setting among decision-makers, physicians, private citizens and patients were studied [29], it was shown that private citizens and patients in general had high expectations regarding what should be offered by the public health care system. These expectations did not correspond with the decision-makers' and physicians' view [29]. In a study, by Hammarström [30] involving politicians, civil servants and health care professionals it was found that the medical ethical principals established by Swedish health care legislation were not much reflected in the decision making process when priorities or budget cuts were being decided upon. Instead of seeing these actors as being insensitive, these results were interpreted as being due to the vague ethical guidelines and the difficult nature of the issues being faced [30]. The justness in the distribution of care mentioned in our study was associated with the fact that the voices of some groups are stronger than others.

Deficient information due to the poor reporting systems were experienced by the HDMs as problematic as it could lead them to make incorrect decisions. One of the purposes of the "Swedish National Study on Aging and Care" [31] was to develop useful descriptions of the older patients needs in the health care system. A study by Mamhidir et al. [32] was conducted in which one of the previously developed systems was applied. Reporting systems can help reduce the problems that lead to ethical difficulties or as mentioned by HDMs, increase them. When referring to the idea of Heidegger, Sartre and Buber regarding the authentic existence, Nerheim [33] wrote about the blindness related to our own inauthentic understanding that focuses on the theories and the moral systems. It is necessary to set our inauthentic understanding within parentheses since theories and moral principles can be misleading. The inauthentic understanding will tell us what the "fact" is, and that might be risky, as it does not include our "life" and could therefore become a hindrance to what can be sensed and understood. In our study it was noticed that the HDMs seemed to externalize the poor reporting systems and refer to them as something out of their control. Sorlie et al. [18,8] have reported on externalization of ethical dilemmas among enrolled nurses and registered nurses working in acute care settings. The enrolled nurses in contrast placed the responsibility for their failure to achieve a good caring standard on the authorities, administrators and health care system and cited in particular a lack of resources [18]. Registered nurses tended to externalise less and seemed to focus more on the responsibility at hand instead of faulting someone else for the shortcomings [8]. With these results in mind, a necessary dialogue between the different levels might reduce the "finger pointing" and improve the elder care system. External placing of responsibility is known to protect against experiences of stress, which could be caused by qualms of conscience [18].

The HDMs' experiences of being in ethically difficult situations were associated with aspects such as their high and exposed position. They felt that the higher up in the hierarchy they were, the more alone they became, especially when situations got difficult. They also expressed feelings of uncertainty. Loneliness and uncertainty are fundamental conditions of life [34], and as such are therefore unavoidable, and it is these states of existence that make a person vulnerable. Henriksen & Vetlesen [35] empathize that when a person assumes heavy responsibility the unpleasant feelings associated with it can be their conscience striving to influence their ethical integrity. The HDMs in our study realized that these feelings were something they had to learn to live with or quit their jobs since they are required to make final decisions and stand by them. They saw their salary as somewhat of a compensation for the unpleasantness. Professionals in the different health care fields of surgery [14] paediatrics [36] and geriatrics [19] are reported to have accepted the inevitability of uncertainty.

It is obvious that the HDMs were experiencing a lack of confirmation, which is in contrast to what some others working in the health care system have experienced [14,37]. Lack of confirmation can negatively affect a person's identity. Social confirmation and recognition from others is needed for the construction of ones identity [38]. Our self image is formed by social confirmation and the lack of it could lead to a breakdown of ones self image and contribute to mistrust [34], which can negatively affect how well we act with others. The risk of becoming scapegoats or being threatened was mentioned in our study as contributing to emotional strain. The occurrences of threats and violence against elected officials have been reported to be 16% at the municipal level and 20% at the county council level [39]. Included in these threats considered to be serious not only against the individual but against the democracy as well were: assault and battery, unlawful threat, slander and insult [39]. Facing these risks expressed the sense of vulnerability.

Living with divided feelings proved ethically challenging when the HDMs' loyalty to their jobs came into conflict with their convictions. Decisions they thought could be detrimental to the older adults gave them a felling of failure. Guilt can be felt if a person does not meet what they feel is required of them in a situation or deny something valuable in their own life [34]. In our study it seems that the HDMs remembered their fallibility in their dealings with other difficult situations. This indicates what Ricoeur [40] calls the memory of ethics, which means that people never can or will forget situations in which they failed to do right or something good. The HDMs felt a loyalty to their job because of the responsibility they had assumed, even when uncomfortable decisions had to be made. This is in line with the reasoning that Lundqvist [41] presented where in some situations an ethical demand confronting an administration can be made less important if a legitimate authority had given directives concerning it. Individuals that are attended to in a system can feel a strong commitment to it and the authority represented in it causes a willingness to obey [42]. However, it is important to not blindly obey others but base actions on ones own ethical judgements [35]. Possible motives behind the HDMs loyalty to their positions could be the desire to bring about good elder care or to benefit their own self-interests.

In our study, different ethical dilemmas and the meaning of being in ethically difficult situations related to elder care have been revealed by the HDMs. This confirms the idea of Lindseth [9] that both an action and relational ethics perspective persists simultaneously and are closely related. This is so even though the HDMs are not directly involved with the patients or the professionals. As leaders it disturbs them when they receive reports or hear of not only bad incidents occurring in elder care but also of bad relationships. When this occurs these issues are up close and personal, they are touched by them, their feelings are moved and they become directly involved. They also expressed feeling uncertain as to whether their decisions would lead to good care. It is therefore understandable that the HDMs deal with questions that reflect both perspectives such as "What should I do" as well as "How do I fulfil my role [9]. This ethical theory [9] is useful for illustrating the complexity of the ethical challenges and that ethics concerns everyone, caregivers as well as HDMs.

Health administrators and politicians have been viewed as having little understanding for the demands expressed by staff in acute care [18] and have been reported as probably cold and cynical [12]. In our study, it is reasonable to believe that the HDMs' experiences of being ethically challenged concerns their feelings that important issues and needs are at stake in elder care as well as for themselves.

The crux of the ethical challenges seems to be related to the HDMs having a covenant with older patients and society to provide good care and that this care is governed by the limited budgets of the different health care organisations. Bakken et al. [43] stressed that the rhetoric regarding the welfare state at the national level, including the health care sector can be experienced as being almost unlimited. The welfare state ambitions are executed at the local political level and the disparity between the ambitions and available recourses systematically creates ethical dilemmas [43]. The HDMs, in our study requested a public debate addressing what can be expected from and offered by the national public health care systems. This underlines their uncertainty about how to deal with troublesome situations including reports of problems in elder care. A wish for some support for making decisions or maybe some relief by sharing these difficult issues with others might be sought. It might also reflect what Thompson [25] calls restoring responsibility in health care. The fundamental key issue of trust between individuals must be transferable into the character of the health care system or organisations. Studies have addressed the concept called "organisational ethics" in which, for example, the issues related to ethical conflicts at different levels and by different professionals in the organisation are revealed. The management is required to make sure that the necessary prerequisites are provided to ensure that the structures and processes enable dialogues concerning ethical issues and behaviours within a health care organisation [44-46].

Our paper provides further insight into the ethical dilemmas and ethical challenges met at the HDM level, which is important since their decisions affect many stakeholders in elder care. According to our results it seems that ethical discussions do not have a high priority on the HDMs agenda. The distance between the patients, professionals and HDMs may affect that situation. Our results can be used to stimulate discussions between HDMs and health care professionals concerning ways of dealing with ethical issues and the necessity of structures that facilitate dealing with them. Ethical reflections will probably have an impact on the trust in the caring system. Future research is suggested that studies how situations affect people when loyalties to a position come into conflict with personal convictions. Since ethical dilemmas that confront all levels of health care organisation will persist, the concept of organisational ethics also needs further research. Our paper provides further insight into the ethical dilemmas and ethical challenges met by high level decision-makers', which is important since the overall responsibility for elder care that is also ethically defensible rests with them. They have power and their decisions affect many stakeholders in elder care. Our results can lead to stimulating discussions between high level decision-makers and health care professionals concerning ways of dealing with ethical issues and the necessity of structures that facilitate it. Even if the high level decision-makers have learned to live with the ethical challenges that confronted them, it was obvious that they were not free from feelings of uncertainty, frustration and loneliness. Vulnerability was revealed regarding themselves and others. Their feelings of failure indicated that they felt something was at stake for the older adults in elder care and for themselves as well, in that there was the risk that important needs would go unmet.

## Methodological consideration

When using a phenomenological hermeneutic method it is important that the participants are eager to talk about the area of concern [16]. Our sampling procedure that consisted of drawing names from a list until the desired number of willing participants was achieved was considered an appropriate method of securing eager participants. Careful consideration was given regarding the number of participants since with too many, there is a risk for data redundancy. Data redundancy was also prevented since the data was analysed as ethical dilemmas or the meaning of being in ethically difficult situations. The interviews were performed within two counties decreasing the risk for participant recognition. In order to obtain as many rich narratives as possible about the HDMs experiences of ethical dilemmas, the interviewees were not interrupted during their narrative reflection. In interviews that focus on difficulties, an indirect idea of what 'a' good life is or what 'the' good is might arise through descriptions of what is missing or what is at stake [47]. The interviews gave the HDMs a possibility to talk about what was important to them. Interviews can be used to obtain information about the meaning of lived experiences from particular situations [48]. Analyzing narratives of lived experiences can be a useful method of providing new insights [49]. With all the authors taking part in the analyses the credibility of the study is increased. The phenomenological hermeneutic method seemed to be useful for this study since the aim was to illuminate lived experiences and this method is developed for such aims [16]. However, the naïve reading of the text will determine if it is possible to perform such an analysis. If a text consists of more descriptions than lived experiences, then a latent content analysis is preferable. Our text proved to be very rich, well detailed and consisted of many thoughts and feelings, which made this method suitable. The method does not however allow comparison between groups in the quantitative sense but the narratives can reveal differences between the groups from the interview text. It revealed possibilities of interpreting lived experiences and can provide a basis for reflection and discussion concerning the ethical dilemmas confronted by HDMs related to elder care. Consequently the results of this study cannot be generalized, but are credible if persons with similar experiences can recognize the descriptions or the interpretations [50] and if these can be transferred into similar situations [19].

## Competing interests

The author(s) declare that they have no competing interests.

## Authors' contributions

AGM participated in the design of the study, carried out the interviews, participated in the analysis, completed and approved to the final manuscript.

MK and VS participated in the design of the study, read the interviews, participated in the analysis, helped to complete and approved the final manuscript.

## Pre-publication history

The pre-publication history for this paper can be accessed here:


